# Progesterone, estradiol, arachidonic acid, oxytocin, forskolin and cAMP influence on aquaporin 1 and 5 expression in porcine uterine explants during the mid-luteal phase of the estrous cycle and luteolysis: an *in vitro* study

**DOI:** 10.1186/s12958-015-0004-5

**Published:** 2015-02-18

**Authors:** Agnieszka Skowronska, Patrycja Młotkowska, Bartosz Wojciechowicz, Stanisław Okrasa, Soren Nielsen, Mariusz T Skowronski

**Affiliations:** Department of Human Physiology, Faculty of Medical Sciences, University of Warmia and Mazury in Olsztyn, Olsztyn, Poland; Department of Animal Physiology, Faculty of Biology and Biotechnology, University of Warmia and Mazury in Olsztyn, Olsztyn, Poland; Department of Health Science and Technology, Faculty of Medicine, Aalborg University, Aalborg, Denmark; Institute of Veterinary, Poznań University of Life Sciences, Poznań, Poland

**Keywords:** Aquaporins, Uterus, Estrous cycle, Pig, *in vitro* study

## Abstract

**Background:**

The cell membrane water channel protein, aquaporins (AQPs), regulate cellular water transport and cell volume and play a key role in water homeostasis. Recently, AQPs are considered as important players in the field of reproduction. In previous studies, we have established the presence of AQP1 and 5 in porcine uterus. Their expression at protein level altered in distinct tissues of the female reproductive system depending on the phase of the estrous cycle. However, the regulation of aquaporin genes and proteins expression has not been examined in porcine uterine tissue. Therefore, we have designed an *in vitro* experiment to explain whether steroid hormones, progesterone (P4) and estradiol (E2), and other factors: oxytocine (OT), arachidonic acid (AA; substrate for prostaglandins synthesis) as well as forskolin (FSK; adenylate cyclase activator) and cAMP (second messenger, cyclic adenosine monophosphate) may impact AQPs expression.

**Methods:**

Uterine tissues were collected on Days 10–12 and 14–16 of the estrous cycle representing the mid-luteal phase and luteolysis. Real-time PCR and Western blot analysis were performed to examine the expression of porcine AQP1 and AQP5. Their expression in the uterine explants was also evaluated by immunohistochemistry.

**Results:**

The results indicated that uterine expression of AQP1 and AQP5 potentially remains under control of steroid hormones and AA-derived compounds (*e.g.* prostaglandins). P_4_, E_2_, AA, FSK and cAMP cause translocation of AQP5 from apical to the basolateral plasma membrane of the epithelial cells, which might affect the transcellular water movement (through epithelial cells) between uterine lumen and blood vessels. The AC/cAMP pathway is involved in the intracellular signals transduction connected with the regulation of AQPs expression in the pig uterus.

**Conclusions:**

This study documented specific patterns of AQP1 and AQP5 expression in response to P4, E2, AA, FSK and cAMP, thereby providing new indirect evidence of their role in maintaining the local fluid balance within the uterus during the mid-luteal phase of the estrous cycle and luteolysis in pigs.

## Background

Since the discovery of aquaporins (AQPs), water channel proteins, a high rate of transcellular water flow is believed to be mediated by these specialized protein transporters. Recently, AQPs have become considered to be important players in the field of reproduction, see reviews [1, 2]. Several AQP isoforms are expressed in the female reproductive tissues: ovary, uterus, placenta, amnion and chorion cytotrophoblasts [3–10]. Their specific expression pattern suggests that they participate in water movement between the intraluminal, interstitial and capillary compartments. Further studies have demonstrated that AQPs are also involved in endometrial development, cell migration and invasion [11]. Aquaporin 1 and 5 are water selective and belong to the classical AQP family [12]. AQP1 is a 28-kDa water channel protein expressed in the endothelial and epithelial cells of many tissues, increasing water permeability of the cell membrane. AQP5 has mainly been localized in apical plasma membranes of various secretory glands [13]. The important role of AQP5 in water homeostasis is evidenced by AQP5-null mice which have reduced saliva secretion [14].

The expression of AQPs in uterine tissue was first described by Li et al. [15], who confirmed the presence of AQP1 transcript in the human uterus. Afterwards, Li et al. [16] demonstrated that AQP1 mRNA expression in the rat uterus is up-regulated by estradiol. Accumulating evidence indicated that ovarian steroids can affect the expression of several AQPs in the reproductive system, including the uterus [17, 18]. The presence of AQP1, 2 and 5 has also been studied throughout the estrous cycle in bitches [19]. Very recently, Klein et al. [20] showed uterine mRNA expression of 12 different AQPs subtypes in endometrium of cyclic and pregnant mares. Moreover, it was demonstrated that cAMP is involved in up-regulation of some AQPs in a variety of cell types [9,21–24]. The presence of AQP1 in human endometrial blood vessels indicates its involvement in the regulation of edema, and in the regulation of angiogenesis [25] as well as pathological processes related to ovulatory uterine bleeding in women [26].

Studies on pigs suggest a functional collaboration among diverse AQPs within the uterus during different phases of the estrous cycle and early pregnancy [27]. It has been shown that AQP5 is localized in myometrial and epithelial cells of the uterus, but AQP1 in uterine endometrial and myometrial blood vessels [7, 27]. Their expression at a protein level was also altered in distinct tissues depending on the phase of the estrous cycle and the stages of early pregnancy. However, the regulation of aquaporin genes and protein expression has not been examined in porcine uterine tissue. Therefore, we have designed an *in vitro* experiment to explain whether steroid hormones, progesterone (P_4_) and estradiol (E_2_), and other factors: oxytocine (OT), arachidonic acid (AA; substrate for prostaglandins synthesis) as well as forskolin (FSK; adenylate cyclase activator) and cAMP (cyclic adenosine monophosphate; second messenger) may have impact on the AQPs expression. Consequently, the primary aim of this study was; (i) to examine the changes in AQP1 and AQP5 at mRNA and protein levels in porcine uterine explants in the presence of P_4_, E_2_, OT, AA, FSK and cAMP; and then (ii) to describe the effect of tissue exposition duration to the experimental factors on the AQPs expression; (iii) to compare their expression in the uterine explants, representing the mild-luteal phase of the estrous cycle and luteolysis; (iv) to determine the localization of AQP1 and 5 in uterine explants after the treatments. This study provided additional information concerning factors potentially responsible for water homeostasis in porcine uterus during the mid-luteal phase and luteolysis in the pig.

## Methods

### Experimental animals and collection of uterine tissue

All experiments were performed in accordance with Animal Ethics Committee, University of Warmia and Mazury in Olsztyn, Poland (AEC approval No. 66/2010/DTN). Tissue samples were recovered from mature cross-bred gilts (Large White × Polish Landrace) on Days 10–12 (n = 5 per group) during the mid-luteal phase and on Days 14–16 (n = 5 per group) of the estrous cycle (the stage of luteolysis). Gilts were observed daily for estrous behavior, and they were used in the study during their third consecutive normal estrous cycle. The animals were slaughtered at a local abattoir on Days10-12, corresponding to the period with increased plasma progesterone (P_4_) concentration, or Days 14–16, the period of decreasing plasma P_4_ concentration and luteal regression, of the estrous cycle. Additionally, stage of the cycle was verified by utero-ovarian morphology [28]. Uteri were placed immediately in ice-cold phosphate-buffered saline (PBS) supplemented with 100 IU/ml penicillin (Polfa, Poland) and 100 μg/ml streptomycin (Polfa, Poland) and transported to the laboratory on ice within 1 to 1.5 h for *in vitro* tissue culture.

### Preparation and incubation of uterine slices

Sections of the middle part of uterine horn collected from pigs were opened longitudinally on the mesometrial surface. Uteri were washed three times in sterile PBS then carefully cut into small pieces (400 mg weight) and then washed three times in medium M199 (Sigma, USA). Individual uterine slices were placed in culture vials containing 2 ml Medium 199 supplemented with 0.1% BSA (Sigma), 20 μg nystatin (Sigma) and 20 μg gentamicin (Krka, Novo Mesto, Slovenia) and then preincubated *in vitro* under atmosphere of 95% O_2_ and 5% CO_2_ at 37°C for 18 h. After preincubation, the culture medium was replaced with fresh medium, and the explants were treated with vehicle (control) or P_4_ (10^−5^ M; Sigma), E_2_ (10^−9^ M; Sigma), OT (10^−7^ M; Sigma), AA (10^−5^ M; Sigma), FSK (10 μg/mL; Sigma) and cpt-cAMP analog (200 μM; Sigma) and incubated for an additional 3 or 24 h. All treatments were performed in triplicates. Furthermore, uterine tissue explants were snap-frozen in liquid nitrogen (for RNA and protein extraction) and stored at −80°C until further use.

### Total RNA isolation, cDNA synthesis and quantitative real-time polymerase chain reaction analysis

Total RNA was extracted, using the total RNA Prep Plus kit (A&A Biotechnology, Gdansk Poland) according to the manufacturer`s protocol, from uterine explants collected after *in vitro* culture. Total RNA quality and quantity were determined with spectrophotometry (NanoDrop ND-1000, Thermo Scientific, Wilmington, DE, USA). Total RNA samples were transcribed to cDNA using an Enhanced Avian HS RT-PCR Kit it (Sigma) and a mix of dNTPs and random hexamers as primers. Real-Time PCR was performed in duplicate for each sample using a 7300 Real-Time PCR system and SYBR®Green PCR Master Mix (Life Technologies, Grand Island, NY, USA). Real-Time PCR reaction included 12.5 μl SYBR Green PCR master mix, 1 μM forward and reverse primers each and reverse transcribed cDNA (3.5 μl of diluted RT product) supplemented with water to a volume of 25 μl. The conditions of the thermal cycling for each gene were: initial denaturation for 10 min at 95°C, denaturation for 15 sec at 95°C, primer annealing for 1 min at 60°C. Specific primers for *AQP1* and *AQP5* (Table 1) were designed with the Primer Express 3.0 software (Life Technologies) and their specificities were confirmed by comparison of their sequences with the sequence of *AQP1* and *AQP5* deposited in a database and calculation of the statistical significance of the match was performed using the Basic Local Alignment Search Tool (BLAST). For the specificity control, non-template controls and dissociation curve analysis of the amplified products were used for each amplification. The specificity of amplifications was further validated with electrophoresis of the putative amplicons in a 2% agarose gel and, after extraction from gel, automated sequencing using 3730xl DNA Analyzer (Life Technologies). Levels of gene expression were calculated using the ΔΔ Ct method and normalized using the geometrical means of reference genes expression levels, glyceraldehyde 3-phosphate dehydrogenase (*GAPDH*) and *18S rRNA*.

**Table 1:** **Primer pairs used in the study**

**PCR product**	**Sequence**
*AQP1*	Forward: 5′-CCAGCGAGTTCAAGAAGAAG-3′
	Reverse: 5′-GCGACACCTTCACGTTATC-3′
*AQP5*	Forward: 5′-CTATGAGTCCGAGGAGGATT-3′
	Reverse: 5′-GCTTCGCTGTCATCTGTT-3′
*18SrRNA*	Forward: 5′-GGCTACCACATCCAAGGAAG-3′
	Reverse: 5′-TCCAATGGATCCTCGCGGAA-3′
*GADPH*	Forward: 5′-GACCTCCACTACATGGTCTA-3′
	Reverse: 5′-AAGATGGTGATGGCCTTTC-3′

### SDS-PAGE and Western blot

The tissues were placed in ice-cold dissection buffer (0.3 M sucrose, 25 mM imidazol, 1 mM EDTA in ddH_2_O, pH 7.2) containing 8.4 μM leupeptin and 0.4 mM pefabloc [29]. The tissue samples were homogenized using an ultra Turrax T8 homogeniser (IKA Labortechnik, Staufen, Germany) and centrifuged at 4,000 × g for 15 min at 4°C. The supernatant was diluted in SDS buffer contained a final concentration of 62 mM Tris (hydroxymethyl)-aminomethane, 0.1 M sodium dodecyl sulphate (SDS), 8.7% glycerol, 0.09 mM bromophenol blue and 0.04 M dithiothreitol (DTT), pH 6.8. The protein samples were heated for 5 min at 90°C and stored in refrigerator for further analysis. Total protein amounts were determined with spectrophotometry (NanoDrop ND-1000, Thermo Scientific, Wilmington, DE, USA). The samples warmed up to 37°C were loaded into 12.5% polyacrylamide gels and proteins were separated by electrophoresis. The proteins of studied gels were then electro-transferred onto nitrocellulose membranes (Hybond ECL RPN3032D, Amersham Pharmacia Biotech, Little Chalfont, UK) for 1 h at 100 V. The membranes were blocked with 5% milk in PBS-T (80 mM Na_2_HPO_4_, 20 mM NaH_2_PO_4_, 100 mM NaCl, pH 7.5 and 0.1% vol/vol Tween 20) for 1 h. After washing, the membranes were incubated overnight at 5°C with anti-AQPs or β-actin antibodies. Thereafter, the membranes were washed and incubated with horseradish peroxidase-conjugated goat anti-rabbit IgG secondary antibody (P448, diluted 1:3,000, Dako A/S, Glostrup, Denmark) in PBS-T for 1 h. After washing with PBS-T, the sites of antibody-antigen reaction were visualized with an enhanced chemiluminescence (ECL) system (Amersham Pharmacia Biotech, Little Chalfont, UK) and exposure to photographic film (Hyperfilm ECL, RPN3103K, Amersham Pharmacia Biotech, Little Chalfont, UK). The results of Western blotting were quantified by densitometric scanning of immunoblots with GelScan for Windows ver. 1.45 software (Kucharczyk, Poland). Data were expressed as a ratio of AQP proteins relative to actin protein in OD units.

### Immunohistochemistry

Tissues were fixed by immersion in 4% paraformaldehyde for 24 hr. For preparation of paraffin-embedded tissue sections (4 μm thickness), the tissues were dehydrated in ethanol followed by xylene and finally embedded in paraffin [27]. The staining was carried out using indirect immunoperoxidase. The sections were dewaxed and rehydrated. For immunoperoxidase labeling, endogenous peroxidase was blocked by 0.5% H_2_O_2_ in absolute methanol for 10 min at room temperature. To reveal antigens, the sections were submerged in 1 mM Tris solution (pH 9.0) supplemented with 0.5 mM EGTA and heated in a microwave oven. After the treatment, the sections were left for 30 min in the buffer for cooling. Nonspecific binding of IgG was eliminated by incubating the sections in 50 mM NH_4_Cl for 30 min, followed by blocking in PBS supplemented with 1%BSA, 0.05% saponin and 0.2% gelatin. The sections were incubated overnight at 4°C with primary antibodies diluted in PBS supplemented with 0.1% BSA and 0.3% Triton X-100. The sections were rinsed with PBS supplemented with 0.1% BSA, 0.05% saponin and 0.2% gelatin, and then incubated with horseradish peroxidase-conjugated secondary antibody (Dako A/S, Glostrup, Denmark). Labeling was visualized by 0.05% 3,3 diaminobenzidine tetrahydrochloride (DAB). The microscopy was carried out using an Olympus light microscope (BX51, Japan).

### Primary antibodies

Antibodies to AQP1 and AQP5, used in Western blot analysis and immunocytochemistry, were previously characterized, respectively by Terris et al. [30] and Nielsen et al. [13]. All polyclonal antibodies were affinity-purified (SulfoLink Kit, Pierce, Rockford, IL). Moreover, the anti-β-actin antibody was used (cat. no. A2066; Sigma-Aldrich, St Louis, MO). In our previous study, we demonstrated that anti-AQP1 and anti-AQP5 antibodies preincubated with the immunizing peptide prevented labeling in the pig uterus [7]. In addition, immunoglobulins from non-immunized rabbit were used as a negative control.

### Statistical analysis

All numerical data were analyzed by one-way ANOVA and least significant difference (LSD) post hoc test and reported as the means ± S.E.M. from five separate experiments (pigs), each performed in duplicates. Statistical analyses were performed using the Statistica program (StatSoft Inc., Tulsa, USA). Values for p < 0.05 were considered statistically significant.

## Results

### AQP1 mRNA expression in porcine uterine explants

**Figure 1:**
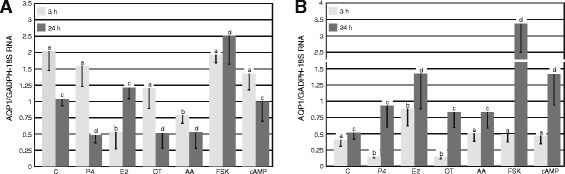
***AQP1*** **mRNA expression in porcine uterine explants.** The expression of *AQP1* mRNA in the pig uterine tissue harvested on **(A)** Days 10–12 (n = 5) and **(B)** Days 14–16 (n = 5) of the estrous cycle after treatment with progesterone (P_4_; 10^−5^ M), estradiol (E_2_; 10^−9^ M), oxytocin (OT; 10^−7^ M), arachidonic acid (AA; 10^−5^ M), forskolin (FSK; 10 μg/mL) and cyclic-AMP (cAMP; 200 μM) for 3 and 24 h. The data are presented as the mean values ± SEM of *AQP1* expression in relation to expression of *GAPDH* and *18 sRNA*. Different letters (a,b,c,d) indicate significant differences (p < 0.05) between each treatment and respective control for 3- (a,b) or 24-h (c,d) incubations.

The control abundance of *AQP1* transcript harvested in the uterine tissue from mid-luteal phase (Days 10–12) was about 4-fold and 2-fold higher (p < 0.05) than on Days 14–16 (luteolysis) during 3-h and 24-h incubation, respectively (Figure 1A and B). In porcine uterine explants from Days 10–12 (the mid-luteal phase) of the estrous cycle, *AQP1* mRNA expression significantly (p < 0.05) decreased after 3-h treatment with E_2_ and AA, in comparison to the respective controls. In turn, longer treatment (24 h) with FSK significantly (p < 0.05) increased *AQP1* mRNA expression, but with P_4_, oxytocin and AA it decreased.

Aquaporin 1 gene expression in porcine uterine tissue explants from Days 14–16 (luteolysis), significantly (p < 0.05) increased after both 3- and 24-h treatment with E_2_ (Figure 1B). In contrast, *AQP1* mRNA expression decreased after 3-h treatments with P_4_ and OT (p < 0.05). Arachidonic acid, FSK and cAMP did not affect *AQP1* mRNA expression during shorter incubation. In turn, an increase in *AQP1* mRNA expression was seen after 24-h treatment with FSK and cAMP (p < 0.05). A particularly strong increase (~7-fold) in AQP1 mRNA expression was noted after 24-h treatment with FSK.

### AQP5 mRNA expression in uterine explants

**Figure 2:**
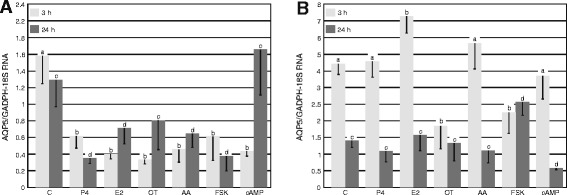
***AQP5*** **mRNA expression in porcine uterine explants.** The expression of *AQP5* mRNA in the pig uterine tissue harvested on **(A)** Days 10–12 (n = 5) and **(B)** Days 14–16 (n = 5) of the estrous cycle after treatment with progesterone (P_4_; 10^−5^ M), estradiol (E_2_; 10^−9^ M), oxytocin (OT; 10^−7^ M), arachidonic acid (AA; 10^−5^ M), forskolin (FSK; 10 μg/mL) and cyclic-AMP (cAMP; 200 μM) for 3 and 24 h. The data are presented as the mean values ± SEM of AQP5 expression in relation to expression of *GAPDH* and *18 sRNA*. Different letters (a,b,c,d) indicate significant differences (p < 0.05) between each treatment and respective control for 3- (a,b) or 24-h (c,d) incubations.

Quantitative expression of *AQP5* mRNA in the uterine explants on Days 10–12 and 14–16 of the estrous cycle is presented on Figure 2A and B, respectively. The content of *AQP5* transcript in uterine explants following control incubation was about 2.5 fold lower after 3-h incubation (p < 0.05) on Days 10–12 of the estrous cycle in comparison to Days 14–16. Such a difference was not observed in the case of longer incubation (24 h). During the mid-luteal phase of the cycle, markedly lower amounts of *AQP5* transcript were noted after treatments with P_4_, E_2_, oxytocin, AA, FSK and cAMP for 3 h as compared to control value (p < 0.05) and remained lower after 24 hours (p < 0.05) except for treatment with cAMP.

Treatment with E_2_ for 3 h of the uterine explants representing luteolysis (Days 14–16) of the estrous cycle significantly increased (p < 0.05) *AQP5* mRNA expression (p < 0.05) but OT and FSK treatments significantly decreased it. Incubation with P_4_, E_2_, OT and AA did not result in any detectable change in porcine uterine explants *AQP5* gene expression after 24-hours incubation. Treatment with cAMP for 24 h significantly decreased *AQP5* gene expression. Conversely, incubation with FSK after 24 h, significantly increased *AQP5* gene expression.

### Protein content of AQP1 in uterine explants

**Figure 3:**
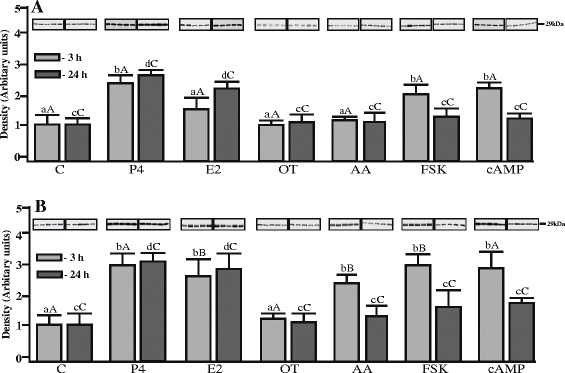
**Protein content of AQP1 in uterine explants.** Semi-quantitative Western blot analysis of AQP1 in homogenates of the pig uterine explants from **(A)** Days 10–12 (n = 5) and **(B)** Days 14–16 (n = 5) of the estrous cycle (each lane represents a sample from 1 pig), after treatment with progesterone (P_4_; 10^−5^ M), estradiol (E_2_; 10^−9^ M), oxytocin (OT; 10^−7^ M), arachidonic acid (AA; 10^−5^ M), forskolin (FSK; 10 μg/mL), and cyclic-AMP (cAMP; 200 μM. Densitometric analysis of the immunoblots was performed and different small letters (a, b,c,d) indicate significant differences (p < 0.05) between each treatment and respective control for 3- (a,b) or 24-h (c,d) incubations. Different large letters **(A, B, C, D)** indicate significant differences between the same treatments for 3- **(A,B)** or 24-h **(C,D)** incubations for different periods (Days 10–12 and 14–16) of the estrous cycle (p < 0.05).

The effects of P_4_, E_2_, oxytocin, AA, FSK and cAMP on AQP1 protein expression in uterine tissue explants harvested during the mid-luteal phase (Days 10–12) and luteolysis (Days 14–16) are shown in Figure 3A and B. The protein expression of AQP1 in porcine uterine explants increased (p < 0.05) after treatment with P_4_ after 3- and 24-h incubation. Estradiol treatment for 3 h did not significantly affect AQP1 protein expression in the tissue from mid-luteal phase, however, after longer incubation (24 h) E_2_ stimulated its expression (p < 0.05). AQP1 protein expression significantly increased after 3-h treatment with FSK and cAMP. In turn, treatment with OT and AA did not influence AQP1 expression. During luteolysis, the content of AQP1 was increased (from 2.5- to 3-fold; p < 0.05) by P_4_, E_2_, AA, FSK and cAMP after 3-h incubation (Figure 3A). After 24-h incubation, only P_4_ and E_2_ significantly stimulated AQP1 expression (p < 0.05), similar to the levels noted after 3-h incubation (Figure 3A). It is noteworthy that the responses of AQP1 to E_2_, AA and forskolin, after short incubation, appeared to be higher on Days 14–16 than on Days 10–12 of the estrous cycle.

### Protein content of AQP5 in uterine explants

**Figure 4:**
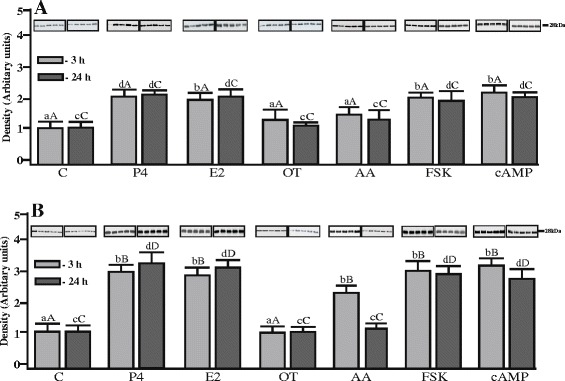
**Protein content of AQP5 in uterine explants.** Semi-quantitative Western blot analysis of AQP5 in homogenates of the pig uterine explants from **(A)** Days 10–12 (n = 5) and **(B)** Days 14–16 (n = 5) of the estrous cycle (each lane represents a sample from 1 pig), after treatment with progesterone (P_4_; 10^−5^ M), estradiol (E_2_; 10^−9^ M), oxytocin (OT; 10^−7^ M), arachidonic acid (AA; 10^−5^ M), forskolin (FSK; 10 μg/mL) and cyclic-AMP (cAMP; 200 μM). Different small letters (a, b, c, d) indicate significant differences (p < 0.05) between each treatment and respective control for 3- (a,b) or 24-h (c,d) incubations. Different large letters **(A, B, C, D)** indicate significant differences between the same treatments for 3- **(A,B)** or 24-h **(C,D)** incubations for different periods (Days 10–12 and 14–16) of the estrous cycle (p < 0.05).

AQP5 expression evaluated by Western blot analysis of in porcine uterus explants from the mid-luteal phase and the luteolysis is shown in Figure 4A and B. Bands of AQP5 protein product of the expected size (28 kDa) were clearly detected in both stages of the cycle. During the mid-luteal phase, uterine AQP5 protein expression significantly (p < 0.05) increased after 3-h and 24-h of treatment with P_4_, E_2_, FSK and cAMP. During luteolysis, a high increase (~3.0-fold) in AQP5 protein expression in the tissue was observed in response to P_4_, E_2_, FSK and cAMP after both 3-h and 24-h incubations (p < 0.05). AQP5 protein content in the uterine tissue was only elevated (~2.5-fold) by 3-h treatment with AA (p < 0.05). In turn, treatment with OT did not influence AQP5 expression in the studied tissue. It is noteworthy that AQP5 protein response to some of the studied factors appeared to be higher on Days 14–16 than on Days 10–12 of the estrous cycle. In particular, during the luteolysis a higher AQP5 protein expression was observed after 3-h treatment with P_4_, E_2,_ FSK and cAMP and remained elevated for 24 hours in comparison to the mid-luteal phase.

### Immunohistochemical localization of AQP1 and AQP5 in uterine tissue explants

**Figure 5:**
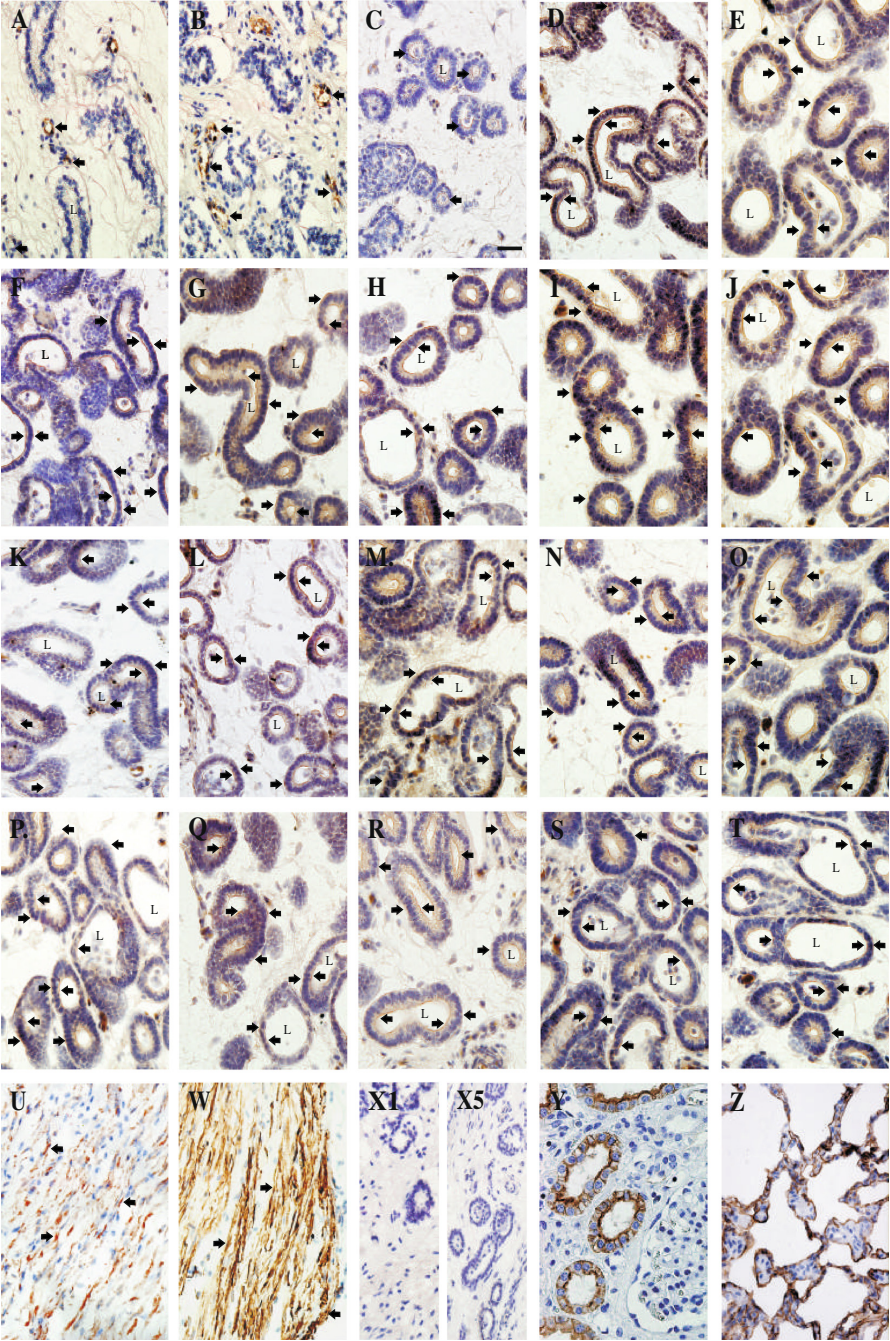
**Immunohistochemical localization of AQP1 and AQP5 in uterine tissue explants.** Immunoperoxidase staining of AQP1 in paraffin-embedded sections of the uterine explants from pigs. Anti-AQP1 antibody labels endothelial cells of the uterine explants (arrows). Both apical and basal plasma membranes exhibited stable AQP1 labeling (**A**/ an example of the staining for the control and **B**/ from the E_2_-treated uterine explants for 24-h on Days 14–16 of the cycle). Immunoperoxidase labeling of AQP1 in the pig kidney cortex (**Y**/ positive control). The labeling is seen in both of the apical and basolateral plasma membrane in proximal tubule cells. AQP5 antibody stains epithelial cells of the uterine explants (arrows). The labeling is seen only in the apical plasma membranes of the epithelial cells (**C**/ an example of the staining for the control on Days 14–16 of the cycle). Continuously, 3- and 24-h treatments on Days 10–12 and 14–16 of the estrous cycle of the tissue explants with P_4_ **(D-E and** **F-G)**, E_2_ **(H-I and** **J-K)**, FSK **(L-M and** **N-O)** and cAMP **(P-Q and** **R-S)**, respectively, prominent AQP5 labeling is seen in both the apical and basolateral plasma membranes of the epithelial cells. A similar pattern of AQP5 labeling is also seen after 3-h treatment with AA on Days 14–16 of the estrous cycle **(T)**. AQP5 antibody also stains smooth muscle cells (**U**/ an example of the staining for the control and **W**/ from the E_2_-treated uterine explants for 24-h on Days 14–16 of the cycle). The anti-AQP5 labels apical membrane of type I pulmonary epithelial cells of the pig (**Z**/ positive control). No staining was observed with using non-immune immunoglobulins (negative controls: **X1** – AQP1 and **X5** – AQP5 controls). L – lumen. Bar = 50 μm.

In the tissue sections of the pig uterine explants representing the mid-luteal phase and luteolysis, immunoperoxidase labeling for AQP1 was associated with uterine endothelial cells (Figure 5A and B). Both apical and basal plasma membranes exhibited stable AQP1 labeling -without intracellular changes in localization - after 3-h and 24-h incubation with all examined factors (Figure 5A and B). However, expression of AQP1 increased after 3- and 24-h tissue exposition to P_4_ and E_2_ on Days 10–12 and 14–16 as well as after 3-h incubation with FSK and cAMP on Days 10–12, and AA, FSK and cAMP on Days 14–16 of the estrous cycle. As a positive control, AQP1 labeling was seen in the apical and basolateral plasma membrane in the proximal tubule cells of the pig kidney (Figure 5Y).

In turn, in sections of the explants treated with examined factors, immunoperoxidase staining for AQP5 was associated with uterine epithelial (Figure 5C) and smooth muscle cells (Figure 5U and W). Moreover, AQP5 labeling in the uterine epithelial cells was more intensive in the apical plasma membrane after 3- and 24-h treatment with OT, AA on Days 10–12 and OT as well as 24-h treatment with AA on Days 14–16 than those observed in respective controls. Following 3- and 24-h treatments of the tissue with P_4_, E_2_, FSK and cAMP on Days 10–12 and 14–16 of the estrous cycle, prominent AQP5 labeling was seen in both the apical and basolateral plasma membranes of the epithelial cells (Figure 5D-S). A similar pattern of AQP5 labeling was seen after 3-h treatment with AA on Days 14–16 of the estrous cycle (Figure 5T). In the smooth muscle cells, in contrast to the epithelial cells, changes in AQP5 localization within the cell membranes were not observed in response to applied treatments (Figure 5U-W). As a positive control, AQP5 antibody noticeably stained (Figure 5Z) the apical plasma membrane of the type I pulmonary epithelial cells of the pig.

## Discussion

In the present study, we have demonstrated that two isoforms of *AQPs* (*1* and *5*) mRNAs are expressed in the porcine uterine explants in both studied periods. In our previous study, performed with the use of immunohistochemistry and Western blot, AQP1 and 5 were clearly detected in untreated uterine tissue on Days 10–12 and 14–16 of the estrous cycle. In cyclic gilts, endometrial and myometrial expression of AQP1 did not change significantly, in turn endometrial AQP5 protein expression was significantly higher on Days 14–16 than on Days 10–12 of the cycle [7]. In the present study, we aimed to further investigate, using an *in vitro* system, whether these AQPs are regulated by steroid hormones, cAMP, forskolin, arachidonic acid or oxytocin in porcine uterine tissue at mRNA and protein levels.

In the present study, steroid hormones (P_4_ and E_2_) differentially influenced the expression of AQP1 and 5 in porcine uterine tissue under *in vitro* conditions. *AQP1* gene expression was down-regulated by E_2_ treatment for 3-h during the mid-luteal phase. Furthermore, P_4_ inhibited *AQP1* mRNA gene expression during luteolysis, after short incubation (3 h). Steroid hormones (P_4_ and E_2_) increased AQP1 protein expression during both studied stages of the cycle and incubations. Moreover, E_2_ more effectively stimulated AQP1 protein expression on Days 14–16 than on Days 10–12 of the cycle. In turn, *AQP5* gene expression was also down-regulated after treatments with E_2_ and P_4_ (3 h and 24 h) during the mid-luteal phase, but up-regulated by E_2_ during luteolysis (3 h). As in the case of *AQP1*, longer incubation with steroids down-regulated *AQP5* mRNA expression during the mid-luteal phase. Interestingly, the basic (control) level of *AQP1* transcript was higher (after 3- and 24-h incubations) in the mid-luteal phase (2.07 ± 0.61 and 1.03 ± 0.09, respectively) than during luteolysis, but in the case of *AQP5* (after 3-h incubation) the relationship was inversed (1.59 ± 0.35 and 4.48 ± 0.71, respectively). At the protein level, the changes of AQP1 and 5 expression in response to E_2_ and P_4,_ were not in full agreement with those noted for mRNAs.

Our findings concerning the E_2_ effect on AQP expression are comparable to those reported by Li et al. [16] and Kobayashi et al. [31], who studied this steroid action on the uterine expression of AQP1 in rat and AQP5 in mouse, respectively. In addition, Kobayashi et al. [31] revealed the presence of functional estrogen response element in AQP5 promoter regions which suggests the possibility of direct action of estrogens on AQP5 expression. Richard et al. [5] found increased *AQP1* mRNA expression in mice myometrium in response to estrogen, but AQP5 expression was induced by estrogen only in progesterone-primed uteri. In several studies, the role of P_4_ alone or in combination with estrogens in controlling AQP expression in uterus has been confirmed. Lindsay and Murphy [6] reported increased expression of AQP5 in uterine epithelial cells by progesterone alone and in combination with estrogen. The same authors [18] also noted progesterone-dependent expression of AQP5 and AQP1 in the rat uterus; AQP5 in glandular epithelium and AQP1 in the inner circular layer of myometrium. Aralla et al. [19] demonstrated coincidently elevated expression of AQP5 in the apical plasma membrane of uterine epithelial cells with increased concentrations of P_4_ in plasma. Furthermore, in the mouse uterus exogenous estrogen strongly up-regulated the expression of AQP2, without any effect on AQP5 [17]. Very recently, the presence of mRNA for all *AQPs* (*AQP0* to *12*) has been shown in equine endometrium, while the Western blot analysis confirmed protein expression of AQP0, 2 and 5 [20]. To the contrary, progesterone treatment of anoestrus mares did not enhance the expression of AQPs, indicating that factors other than progesterone or some factors in addition to progesterone are required for the up-regulation of certain AQP subtypes. The above observations are not entirely consistent, the discrepancies seem to result from different animal models and/or experimental protocols applied in the study. For example, Jablonski et al. [17] used ovariectomized and hormonally treated mice, but in the present study porcine uterine explants were used. In turn, the lack of a full relationship – observed in the present study – between the concentration of gene transcripts and respective proteins may result from differentiated stability of mRNAs and/or proteins as well as from independent regulation of transcription, posttranscriptional processes or translation and functioning feedbacks, *i.e.* high protein concentration may suppress mRNA synthesis. Moreover, steroids themselves may alter the stability of mRNA [32]. It is also noteworthy that the processes of transcription and translation are not equally efficient.

The uterus is a target organ for ovarian steroid hormones and undergoes marked changes during the estrous cycle, including: tissue expansion (by 40-60%) and an increase in uterine gland activity during luteal phase [33], hyperemia [34] as well as increased capillary permeability [33]. These changes require increased synthesis of AQPs. In the present study, as documented by Western blot analysis, P_4_ and E_2_ appeared to be effective in controlling AQP1 and AQP5 expression in pigs, suggesting their crucial role in this regulation during the mid-luteal phase of the estrous cycle and luteolysis.

Arachidonic acid is metabolized in the uterus to prostaglandins (PGE_2_ and PGF_2alpha_), which are involved in many reproductive activities including luteolysis, maternal recognition of pregnancy, endometrial gene expression and conceptus development [35–37]. Prostaglandin synthesis is thus dependent upon accessibility of AA [38] and the activity of enzymes involved in its metabolism [39]. In our studies, its potential effect on AQP1 and 5 expression in uterine tissue of cyclic pigs was tested. The expression of *AQP1* and *5* mRNAs were similarly down-regulated by AA during the mid-luteal phase (after 3- and 24-h exposition). Nevertheless, the expression of both AQPs at the protein level was stimulated only during luteolysis in response to shorter exposition to AA. This well corresponds with physiological situation, since PGF_2alpha_ is released by endometrium (epithelial and stromal cells) [40] in a pulsatile manner to cause corpus luteum regression [41]. Thus, our data indirectly indicate that prostaglandins may exert regulatory effects on AQP1 and 5 in uterine tissue. Studies performed by Zelenina et al. [42] have confirmed the interaction of PGE_2_ with AVP in the regulation of AQP2 in the rat renal medulla_._ Nevertheless, the potential involvement of prostaglandins in the regulation of AQPs in the pig uterus requires explanation in further experiments.

Oxytocin is one of the key hormones implicated in controlling the uterine functions. Among others, it affects phosphoinositide hydrolysis [43], expression of COX-2 in uterine tissue [37] and regulates PGF_2alpha_ secretion [43]. The main reason for investigating the effect of oxytocin on AQP1 and AQP5 expression was that pig endometrium secretes oxytocin [44] and possesses its receptors [45]. In the uterus, the concentration of OT receptor in the endometrium changes during the estrous cycle. It has been reported that in the middle luteal phase of the cycle, the density of OT receptor increases in the endometrium and myometrium [46]. In the present experiments, OT decreased *AQP1* and *AQP5* mRNA expression (after 3- and 24-h incubations), without visible changes in protein content. In the pig during luteolytic period, oxytocin is responsible for pulsatile secretion of PGF_2alpha_ and contractions of myometrium [47]. It might be hypothesized that under physiological conditions, inhibitory action of OT on *AQP1* and *5* uterine expression at a transcriptional level is connected with remodeling of endometrial tissues taking place at the end of the luteal phase [33]. Very recently, Ducza et al. [48] demonstrated increased *AQP2* mRNA, but reduced *AQP5* mRNA expression by OT in the rat uterus on Day 18 of pregnancy. Since the role of oxytocin, similar to PGs, in the regulation of AQPs expression and uterine fluid balance, so far is not sufficiently defined, therefore further work is needed to better delineate it.

Previous studies confirmed that intracellular signaling pathway consisting of adenylate cyclase (AC) and cAMP may be involved in the regulation of AQP expression [21–23]. In the present study, the effects of cAMP and forskolin (AC activator) on AQP1 and 5 in the uterine tissue were tested. The treatment with cAMP did not cause striking changes in expression of studied *AQP* mRNAs in the uterine tissue; only increased *AQP1* expression on Days 14–16 (after 24 h) and decreased *AQP5* on Days 10–12 (after 3 h) and Days 14–16 (after 24 h) of the estrous cycle. In a different experimental model, Wang et al. [22] failed to see any changes in the expression of *AQP1, 8* or *9* genes in human amnion-derived WISH cells after *in vitro* treatment with cAMP analog. In our studies, the opposite reaction of *AQP5* mRNA versus protein in response to cAMP is complicated. This discrepancy may result from the regulatory mechanism functioning at the transcription and translation levels, as discussed earlier and requires an explanation in further studies. The effects of forskolin on *AQP1* and *5* mRNAs were very similar to those observed in response to cAMP; i.e. stimulation of *AQP1* and mostly inhibition of *AQP5* (except stimulation on Day 14–16 after 24 h incubation). At the protein level, the expression of AQP1 and AQP5 was up-regulated by forskolin, as in response to cAMP. Studies performed with the use of different cells/tissues [9, 22, 23, 49] or cell lines [21, 23, 24] also confirmed the stimulatory effect of cAMP and/or forskolin on the expression of AQP1 [24], AQP5 [23] and AQP3, 8 and 9 [9, 21, 23, 49]. Furthermore, AQP1 appeared to be up-regulated by arginine vasopressin and cAMP analogue in trophoblast cells [24]. On the basis of our study, it might be concluded that the AC/cAMP pathway participates in the regulation of AQP5 expression in the uterine tissue. However, the engagement of the AC/cAMP pathway, particularly in the regulation of AQP1 expression in this tissue during the estrous cycle remains to be elucidated.

The changes in cellular localization of AQP1 and AQP5 in response to the studied factors, visualized by immunohistochemistry, are particularly interesting (Figure 5). In the uterine tissue, localization of AQP1 was predominantly associated with apical and basal membranes of endothelial cells, but AQP5 with apical membranes of epithelial cells. It is noteworthy that steroid hormones (P_4_ and E_2_), cAMP and forskolin caused an emergence of AQP5 in basolateral membrane of the epithelial cells during both studied periods. It might be thus hypothesized, that these changes are connected with potentially bidirectional transcellular water movement through uterine epithelial cells. Garcia et al. [50] indicated that cAMP may induce insertion of AQP8 within intracellular vesicular structures and translocation to plasma membranes in the rat hepatocytes. Further experiments are necessary to further explain the role of AQPs in uterine water balance in cyclic gilts.

## Conclusions

The present studies delineated the specific AQP1 and AQP5 expression patterns in response to P_4_, E_2_, AA, FSK and cAMP and indirectly provided novel evidence for the role of AQP1 and AQP5 in maintaining local fluid balance within the uterus during mid-luteal phase of the estrous cycle and luteolysis in pigs. They revealed that: (1) steroid hormones (P_4_ and E_2_), oxytocin and presumably AA metabolites (prostaglandins) exhibit potential for regulation of AQP1 and 5 expression in porcine uterine tissue during the luteal phase; (2) AQPs responses to the studied factors depended on the stage of luteal phase and duration of their action; (3) P_4_, E_2_, AA, forskolin and cAMP cause translocation of AQP5 from apical to the basolateral plasma membrane of the epithelial cells, which might affect the transcellular water movement (through epithelial cells) between uterine lumen and blood vessels; (4) the AC/cAMP pathway is involved in the intracellular signals transduction connected with the regulation of AQPs expression in the pig uterus.
